# Prognostic Impact of Right Ventricular Diastolic Dysfunction in Patients Undergoing Isolated Coronary Artery Bypass Grafting

**DOI:** 10.34172/aim.28830

**Published:** 2025-01-01

**Authors:** Tahereh Davarpasand, Arezoo Zoroufian, Rezvan Ahmadi Roknabadi, Mohammad Sadeq Najafi, Zahra Karimi, Soheil Mansourian, Amirhossein Poopak, Roozbeh Narimani-Javid

**Affiliations:** ^1^Tehran Heart Center, Cardiovascular Diseases Research Institute, Tehran University of Medical Sciences, Tehran, Iran; ^2^Research Center for Advanced Technologies in Cardiovascular Medicine, Cardiovascular Diseases Research Institute, Tehran University of Medical Sciences, Tehran, Iran

**Keywords:** Coronary artery bypass, Prognostic factors, Right ventricular dysfunction

## Abstract

**Background:**

Right ventricular diastolic dysfunction (RVDD) increases the volume load on the right ventricle. We aimed to evaluate the association of RVDD with perioperative outcomes in patients undergoing isolated coronary artery bypass graft surgery (CABG).

**Methods:**

This single-center observational study included all consecutive isolated CABG patients with a left ventricular ejection fraction (LVEF)>40% from May 2022 to May 2023 who were evaluated for RV diastolic function by transthoracic echocardiography. We divided patients into two groups, with and without RVDD, and then compared the two groups in terms of the primary outcomes of the duration of hospitalization, intensive care unit (ICU) stay, and intubation time, and the secondary outcome composed of postoperative in-hospital complications.

**Results:**

Our study found that 49.1% of patients suffered from RVDD, and patients with RVDD had significantly lower systolic blood pressure and were more likely to take angiotensin-converting enzyme inhibitors than those without RVDD. There was no association between RVDD and primary outcomes of hospitalization time (β=-0.01; 95% CI -0.05, 0.04; *P* value=0.717), ICU stay (β=0.01; 95% CI -0.18, 0.17; *P* value=0.984) and intubation time ([β=0.06; 95% CI -0.05, 0.17; *P* value=0.309). However, more postoperative complications occurred in patients with RVDD (90% vs. 85%). After adjustment for confounding factors, RVDD was not independently associated with primary and secondary outcomes.

**Conclusion:**

Preexisting RVDD in CABG patients with LVEF>40% increased postoperative complications but not significantly. More extensive studies are needed to evaluate RV diastolic function before cardiac surgery to identify high-risk patients and optimize their perioperative management.

## Introduction

 Coronary artery bypass graft (CABG) represents the leading cardiac surgical intervention globally, with nearly 400 000 procedures conducted annually.^1^ The majority of evidence concerning post-operative outcomes has predominantly focused on left ventricular (LV) function,^2,3^ with few studies exploring the role of the right ventricle (RV) due to the complexities associated with its geometric shape, which complicates functional assessment. The majority of studies regarding RV function emphasize systolic dysfunction, typically associated with significant left-sided heart failure. Perioperative evaluation of right ventricular filling prior to cardiac surgeries needs to be investigated thoroughly.^4^

 Right ventricular diastolic dysfunction (RVDD) is a condition that affects the filling pressure of the RV during diastole. RVDD can be caused by various factors that increase the pressure or volume load on the RV, including LV dysfunction, valvular heart diseases, ischemic heart diseases, pulmonary embolism, and cardiomyopathies.^5-8^ Previous studies have highlighted preoperative RVDD and the lack of suitable target bypass arteries as independent risk factors for early mortality following CABG in individuals with markedly compromised LV function.^4^ Additionally, another study indicates that preoperative RVDD, female sex, and cardiopulmonary bypass serve as independent risk factors for the onset of postoperative heart failure in patients with coronary artery disease undergoing CABG.^9^

 Given that RVDD manifests before systolic dysfunction,^10^ it is prudent to examine its prognostic significance in individuals with preserved left ventricular ejection fraction (LVEF). Therefore, we aimed to investigate whether RV diastolic function is associated with length of hospital stay (LOS), duration of intensive care unit (ICU) stay, intubation time and the composite of complications in patients with no clinically reduced ejection fraction (LVEF > 40) undergoing isolated CABG.

## Materials and Methods

###  Study Population and Data Collection

 This single-center, observational study was conducted at Tehran Heart Center Hospital. All consecutive isolated CABG candidate patients with no clinically reduced LVEF ( > 40%)^11^ from May 2022 to May 2023 were included and assessed for RV diastolic function before surgery. We excluded patients with recent acute coronary syndrome, severe tricuspid valve regurgitation, LVEF < 40%, respiratory failure due to lung disease, chronic kidney or liver disease, and malignant tumors. For each patient who met the inclusion criteria, the demographics and characteristics were collected, including age, sex, body surface area (BSA), heart failure classification according to New York Heart Association (NYHA), previous myocardial infarction (MI), hypertension (HTN), stroke history, diabetes mellitus (DM), hyperlipidemia (HLP), cigarettes smoking (CS), and history of percutaneous coronary intervention (PCI). Furthermore, intraoperative metrics were documented, encompassing cardiopulmonary bypass duration, aortic clamping duration, and graft number. Demographic, perioperative, and postoperative data were obtained through complete history taking and patients’ health records.

## Evaluation of Cardiac Function

 Patients with diminished diastolic RV function were identified using preoperative transthoracic echocardiography (TTE), which was acquired within a week prior to the index procedure. We used the RV focus view to evaluate the RV function by transthoracic echocardiography (Philips Affinity70C: Andover, USA: probe S5-1) and placed the Doppler probe on the RV inflow.^12^ To assess the RV diastolic function, we used the American Society of Echocardiography/European Association of Cardiovascular Imaging (ASE/EACVI) guideline, which recommends using the tricuspid valve (TV) inflow, E/A ratio, the lateral tricuspid annular tissue Doppler velocity (e’) as parameters. All TTEs were performed by one expert echocardiographist. We also measured the tissue Doppler velocity from the lateral tricuspid annulus and obtained the values of e’, a’, e’/a’, and E/e’.^13^

 Et/At ratio values between 0.8 and 2.1 were considered to indicate normal right ventricular diastolic function. However, diastolic RV dysfunction was determined as the Et/At ratio values less than 0.8, higher than 2.1, or Et/et’ ratio values greater than 6. An Et/At ratio value less than 0.8 indicated impaired relaxation of the RV, while values in the range of 0.8 to 2.1 and an Et/et’ ratio greater than 6 pointed to pseudonormal filling of the RV. An Et/At ratio greater than 2.1 indicated a restrictive type of RV filling.^13^

## Outcomes

 We compared the patients with and without RVDD regarding baseline characteristics. The primary outcome of this study was LOS, duration of ICU stay, and intubation time. A composite of complications, including non-fatal acute myocardial infarction, non-fatal stroke, death (cardiac and all-cause), acute renal failure, embolic events, pleural and pericardial effusion, pack cell infusion, atrial and ventricular arrhythmias, intra-aortic balloon pump (IABP), deep vein thrombosis, major gastrointestinal bleeding, DC shock, and tamponade constituted the secondary outcomes of our study.

## Statistical Analysis

 The R software, version 4.1.2, was used for statistical analysis. Absolute values and percentages were used to present qualitative variables. On the other hand, quantitative variables were presented as mean (standard deviation) or medians/quartiles (25th and 75th percentiles). The Mann-Whitney, independent t-tests, and chi-squared tests were used to compare the two groups. We performed logistic regression with adjustment for confounder variables to determine if RVDD is associated with the primary outcomes.

## Results

###  Baseline Characteristics

 The patients were mostly men (72.4%) with a mean age of 62.25 ± 8.76. A total of 112 (49.12%) patients had RVDD. The prevalence of RV systolic dysfunction in our study was 6%. Patients with RVDD and those without RVDD were not significantly different in age, CS, HTN, MI, NYHA, DM, stroke history, PCI history, HLP, taking beta-blockers and statins, renal failure, BSA, heart rate, and diastolic blood pressure (DBP). Men were significantly more diagnosed with RVDD (*P* = 0.003). Patients with RVDD used ACE inhibitors more than patients without RVDD (*P* = 0.035). Baseline characteristics are summarized in Table 1.

**Table 1:** Baseline Characteristics and Perioperative Variables Compared between Patients with and without Right Ventricular Diastolic Dysfunction

**Characteristic**	**Overall** **(N=228)**	**Without RVDD** **(n=116)**	**With RVDD** **(n=112)**	***P *** Value^*^
Sex (male)	165 (72.4%)	94 (81.0%)	71 (63.4%)	0.003
Age (year)	62.25 (8.76)	61.74 (9.27)	62.77 (8.22)	0.5
Smoking	48 (21.1%)	23 (19.8%)	25 (22.3%)	0.6
Hypertension,	115 (50.4%)	52 (44.8%)	63 (56.2%)	0.085
MI history, n (%)	10 (4.4%)	4 (3.4%)	6 (5.4%)	0.5
NYHA				> 0.9
1	53 (23.2%)	26 (22.4%)	27 (24.1%)	
2	147 (64.5%)	75 (64.7%)	72 (64.3%)	
3	28 (12.3%)	15 (12.9%)	13 (11.6%)	
Diabetes mellitus	80 (35.1%)	39 (33.6%)	41 (36.6%)	0.6
Stroke history	5 (2.2%)	3 (2.6%)	2 (1.8%)	> 0.9
PCI history	15 (6.6%)	8 (6.9%)	7 (6.2%)	0.8
Hyperlipidemia	96 (42.1%)	47 (40.5%)	49 (43.8%)	0.6
Beta-blockers	42 (18.4%)	23 (19.8%)	19 (17.0%)	0.6
Statins	98 (43.0%)	48 (41.4%)	50 (44.6%)	0.6
ACE-I	102 (44.7%)	44 (37.9%)	58 (51.8%)	0.035
Renal failure	8 (3.5%)	5 (4.3%)	3 (2.7%)	0.7
BSA	1.80 (0.20)	1.81 (0.19)	1.79 (0.21)	0.6
SBP	118.99 (9.87)	119.44 (9.64)	118.53 (10.13)	0.2
DBP	72.81 (7.97)	72.93 (8.00)	72.68 (7.97)	0.5
HR; Median (IQR)	70.00 (70.00, 80.00)	70.00 (70.00, 80.00)	70.00 (69.50, 78.50)	0.5
**Echocardiographic parameters**				
LVEF (%)				0.7
45	49 (21%)	22 (19%)	27 (24%)	
50	61 (27%)	31 (27%)	30 (27%)	
55	115 (50%)	62 (53%)	53 (47%)	
60	3 (1.3%)	1 (0.9%)	2 (1.8%)	
Left atrium volume index, cc/m^2^	31 (25, 35)	31 (25, 36)	30 (26, 35)	> 0.9
E/A ratio	0.80 (0.69, 0.95)	0.81 (0.71, 1.06)	0.79 (0.69, 0.92)	0.3
E/e’, cm/s	8.80 (7.20, 10.70)	8.65 (7.20, 10.43)	8.95 (7.42, 10.90)	0.5
TAPSE, (mm)	21.00 (19.00, 23.00)	21.00 (19.75, 24.00)	20.00 (19.00, 22.25)	0.033
s’t, cm/s	11.00 (10.00, 12.00)	11.00 (10.00, 13.00)	11.00 (10.00, 12.00)	0.025
Et/At ratio	1.00 (0.81, 1.20)	1.05 (0.90, 1.26)	0.88 (0.72, 1.10)	< 0.001
e’t, cm/s	8.50 (7.00, 10.00)	10.00 (8.00, 11.00)	7.00 (6.00, 9.00)	< 0.001
e’t/a’t, ratio	0.54 (0.46, 0.68)	0.62 (0.52, 0.71)	0.50 (0.41, 0.59)	< 0.001
Et/e’t, ratio	5.46 (4.40, 6.70)	4.75 (4.25, 5.50)	6.70 (5.22, 7.73)	< 0.001
S-wave hepatic vein, cm/s	46 (36, 64)	46 (34, 65)	46 (37, 62)	0.7
D-wave hepatic vein, cm/s	33 (26, 52)	33 (26, 50)	33 (26, 54)	0.8
TRG, mmHg	24.0 (20.0, 27.0)	24.0 (20.0, 28.2)	25.0 (21.0, 27.0)	0.9
**Perioperative Parameters**				
Graft number; median (IQR)	3.00 (3.00, 4.00)	3.00 (3.00, 4.00)	3.00 (3.00, 4.00)	0.4
Cardiopulmonary bypass duration (min)	91.06 (27.21)	92.71 (27.97)	89.36 (26.42)	0.7
Mechanical Support (day)				0.4
1	215 (94.3%)	110 (94.8%)	105 (93.8%)	
2	3 (1.3%)	2 (1.7%)	1 (0.9%)	
3	4 (1.8%)	3 (2.6%)	1 (0.9%)	
4	2 (0.9%)	0 (0.0%)	2 (1.8%)	
≥ 8	4 (1.6%)	1 (0.9%)	3 (2.7%)	
Aortic cross-clamp time (min)	52.79 (16.97)	53.28 (16.84)	52.29 (17.17)	0.8
Inotrope infusion	68 (29.8%)	29 (33.0%)	39 (27.9%)	0.4

Abbreviations: A, Late transmitral diastolic filling; ACE-I, Angiotensin-converting enzyme inhibitor; a’t, Late diastolic tricuspid annular tissue velocity; At, Late trans tricuspid diastolic filling; e’, Early diastolic mitral annular tissue velocity; E, Early transmitral diastolic filling; e’t, Early diastolic tricuspid annular tissue velocity; Et, Early trans tricuspid diastolic filling; LVEF, Left ventricular ejection fraction; NYHA, New York Heart Association; PCI, Percutaneous coronary intervention; RVDD, Right ventricular diastolic dysfunction; s’t, Systolic tricuspid annular tissue velocity; TAPSE, Tricuspid annular plane systolic excursion; BSA, Body surface area; IABP, Intra-aortic balloon pump; TRG, Tricuspid regurgitation gradient; SBP, Systolic blood pressure; DBP, Diastolic blood pressure; HR, Heart rate. Data is presented by n (%), Mean (SD), or median (IQR). *P < 0.05 is considered significant.

 There was no significant difference between the study groups regarding perioperative parameters, i.e. the number of CABG grafts, cardiopulmonary bypass duration, aortic cross-clamp, intubation time, and inotropes infusion (Table 1).

###  Adverse Effects and Mortality

 Patients experienced various postoperative adverse effects, including non-fatal stroke, acute renal failure, pleural effusion, pericardial effusion, tamponade, new-onset atrial fibrillation, ventricular arrhythmia, deep vein thrombosis, and DC shock. Unfortunately, 7 (3.1%) patients died postoperatively, all of whom were cardiovascular-related: 4 (3.6%) patients with RVDD and 3 (2.6%) patients without RVDD. None of the patients had non-fatal MI, thromboembolic events, or major gastrointestinal bleeding (Table 2).

**Table 2:** Postoperative Complications and All-Cause Mortality

**Complications**^a^	**Without RVDD** **(n=116)** **No. (%)**	**With RVDD** **(n=112)** **No. (%)**
Composite	99 (85)	101 (90)
Non-fatal stroke	2 (1.7)	1 (0.9)
Acute renal failure	1 (0.9)	1 (0.9)
Pleural effusion	29 (25)	17 (15)
Pericardial effusion	18 (16)	14 (12)
Tamponade	2 (1.7)	0 (0)
New onset atrial fibrillation	12 (10)	13 (12)
Ventricular arrhythmia	3 (2.6)	6 (5.4)
Deep vein thrombosis	1 (0.9)	0 (0)
DC shock	0 (0)	1 (0.9)
Pack cell infusion	94 (81)	97 (87)
IABP	4 (3.4)	3 (2.7)
All-cause death	3 (2.6)	4 (3.6)
Cardiovascular death	3 (2.6)	4 (3.6)

IABP, Intra-aortic balloon pump; DC, Direct current; RVDD, Right ventricular diastolic dysfunction. ^a^ Non-fatal MI, thromboembolic event, and major gastrointestinal bleeding were also included in the composite, but their value was zero.

###  Multivariate Analysis

 In an adjusted regression model for crucial times as primary outcomes, RVDD had no association with hospitalization, ICU, or intubation time. Although complications were more frequently observed in RVDD patients, in the crude model and after adjusting for sex and ACE-I, our multivariate analysis showed that patients with RVDD did not have a statistically significantly higher risk of developing postoperative adverse events (Tables 3 and 4).

**Table 3:** Association between RVDD and Primary Outcomes

**Models**^a^	**Beta**	**95% CI**	***P *** Value^*^
RVDD-Hospitalization time	-0.01	-0.05, 0.04	0.717
RVDD-ICU time	0.01	-0.18, 0.17	0.984
RVDD- Intubation time	0.06	-0.05, 0.17	0.309

CI, Confidence interval; RVDD, Right ventricular diastolic dysfunction. ^a^ Regression models have been adjusted for sex and ACE-I. * *P* value < 0.05 is considered significant.

**Table 4:** Association between Right Ventricular Diastolic Dysfunction and the Occurrence of the Composite of Complications

**Model**^a^	**OR**	**95% CI**	***P*** **Value**^b^
Crude model: RVDD	1.58	0.71, 3.63	0.3
Adjusted model^a^	1.49	0.66, 3.49	0.3

OR, odds ratio; CI, Confidence interval; RVDD, Right ventricular diastolic dysfunction. ^a^ Regression adjusted for sex and ACE-I use; * *P* value < 0.05 is considered significant.

## Discussion

 This study evaluated the incidence of RVDD in isolated CABG candidates with LVEF > 40% and its effect on in-hospital complications after surgery. Including patients with LVEF > 40% excluded the confounding role of LV systolic dysfunction. Most previous studies have focused on the systolic function of the RV, which usually develops with severe left-sided heart dysfunction. Despite the evidence that RV diastolic dysfunction develops earlier than its systolic dysfunction, the diastolic function of the RV has received less attention.^14-16^ Therefore, this was the first study to investigate whether RV diastolic function has prognostic value in patients with preserved LV function, which is a more common scenario in CABG candidates. We found that RVDD incidence was 49.1% in the study population, but it was not associated with longer LOS in hospital and ICU, intubation time and postoperative complications such as acute renal failure, pleural and pericardial effusion, pack cell infusion, atrial and ventricular arrhythmias, IABP, and cardiovascular death.

 RVDD also seems to be of prognostic importance in the perioperative settings.^9,15,17^ Sumin et al showed that patients with stable CAD are much more likely to have RV diastolic dysfunction than systolic dysfunction. Additionally, they discovered that the occurrence of RVDD was mostly linked to LV systolic dysfunction and older age.^9^ Several studies have demonstrated that in pulmonary hypertension and a diabetic model, RV diastolic dysfunction can develop before systolic failure.^14,16^

 Diastolic dysfunction is much more prevalent in hypertensive individuals. In this regard, Zhang et al measured regional diastolic dysfunction with pulsed wave tissue Doppler in patients with hypertension and demonstrated that when the disease progressed to a more advanced stage and extended duration, the level of regional diastolic dysfunction expanded, exhibiting a trend from the right ventricular wall to the septum and LV wall.^18^ The wall of the RV is less thick than that of the left ventricle. The compensatory ability of right ventricular wall diastolic function in hypertensive individuals may be less effective than that of the left ventricle and interventricular septum. Therefore, a higher prevalence of hypertension and its treatment in patients with RVDD can be justified, as observed in our study, which showed that the partial diastolic function of the RV in hypertensive patients may appear earlier.

 RV diastolic dysfunction is an essential prognostic factor in assessing ventricular dysfunction in heart failure.^19^ Left-sided heart failure is a recognized etiology of pulmonary arterial hypertension. An elevation in right ventricular afterload due to the onset of pulmonary arterial hypertension owing to persistent pulmonary venous hypertension has typically been regarded as the principal mechanism contributing to right ventricular dysfunction in patients with left-sided heart failure.^20^ Heart failure with preserved ejection fraction (HFpEF) is also likely to cause right heart remodeling and failure.^21^ Hypertrophy and extracellular fibrosis may indicate substantial right ventricular remodeling in HFpEF patients. Consequently, RVDD may indicate early right ventricular involvement in HFpEF patients or signify the shift from a compensated to a decompensated state, exacerbated by the presence of risk factors like hypertension.^22^

 Most previous studies show RV function’s predictive value in cardiac surgery. Maslow et al showed that patients exhibiting abnormal right ventricular systolic and diastolic function and concurrent LV systolic dysfunction had less favorable outcomes following CABG.^5^ Similarly, Lella et al showed that in patients having isolated CABG or valve surgery, abnormal RVEF is a better indicator of long-term cardiac re-hospitalization compared to abnormal LVEF.^6^ Jin et al demonstrated that preoperative RVDD and the absence of appropriate target bypass arteries are independent risk factors for early mortality after CABG in individuals with significantly compromised LV function.^15^

 Additionally, in high-risk patients having major vascular surgery, right ventricular systolic dysfunction has been independently linked to increased rates of postoperative major cardiovascular complications and prolonged hospital stays. Sumin et al showed that RVDD is much more prevalent than systolic dysfunction in individuals with stable coronary artery disease, occurring in 46% and 7.5% of cases, respectively. We reported that 49.1% of patients were diagnosed with RVDD, and the prevalence of RV systolic dysfunction in our study was 6%. The occurrence of RVDD was mostly linked to advanced age and LV systolic dysfunction but not to coronary artery lesions.^9^ Conversely, in our study, RVDD was not associated with age, LV systolic dysfunction, or coronary artery lesions.

 The present study had some limitations. The main limitation of our study is that we included a small sample size from a single center and did not evaluate the long-term outcomes of the patients, such as survival and re-hospitalization. Therefore, we cannot determine whether RVDD impacts the long-term prognosis of CABG patients. Furthermore, the characterization of RV function relied on standard parameters of RV systolic and diastolic function, disregarding advanced techniques such as speckle-tracking RV assessment, three-dimensional RV echocardiography, and CMR. Moreover, we considered a complex composite of complications as the secondary outcomes; these might explain the slightly different results from the rest of the literature in our work. While we adjusted for several known confounders, residual confounding due to unmeasured variables may still be present. These factors should be considered when interpreting the results, and further studies are warranted to validate these findings in broader and more diverse populations. We recommend further multi-centric evaluations with a larger sample size to better understand the effects of RVDD on CABG postoperative outcomes.

## Conclusion

 Although patients with RVDD and LVEF > 40% suffered more composite postoperative complications after CABG, the association was statistically insignificant; therefore, more extensive studies are required to identify the predictive role of RV diastolic dysfunction in postoperative outcomes.
